# Dr. Dennis Buell Wiggins

**Published:** 1905-11

**Authors:** 


Dr. DENNIS BUELL WIGGINS, of Buffalo, died September 23, 1905, aged 83 years. He graduated in Cincinnati in 1846, and had resided in Buffalo upwards of fifty years.

